# A new species of the genus *Calommata* Lucas, 1837, with first description of the male of *Calommata hangzhica* Li & Xu, 2022 from Hunan, China (Araneae, Atypidae)

**DOI:** 10.3897/zookeys.1278.183005

**Published:** 2026-04-28

**Authors:** Fengqin Wu, Wenlong Yan, Guchun Zhou, Xianjin Peng

**Affiliations:** 1 College of Life Sciences, Hunan Normal University, Changsha, Hunan 410081, China ational Navel Orange Engineering Research Center, Gannan Normal University Ganzhou China; 2 School of Life Sciences, National Navel Orange Engineering Research Center, Gannan Normal University, Ganzhou, Jiangxi 341000, China College of Life Sciences, Hunan Normal University Changsha China

**Keywords:** COI, key, morphology, Mygalomorpha, purseweb spiders, taxonomy

## Abstract

A new spider species of the genus *Calommata* Lucas, 1837 is described from China: *C. yueluensis***sp. nov**. (♂♀). The male of *C. hangzhica* Li & Xu, 2022 is described for the first time. Detailed descriptions, photographs of the habitus and copulatory organs, as well as the COI gene confirming that the male belongs to *C. hangzhica* are provided. The distribution map and taxonomic key to Chinese *Calommata* species are presented.

## Introduction

Atypidae Thorell, 1870 is a small spider family currently comprising 61 species in three genera (World Spider Catalog 2026). The genus *Calommata* includes 16 species, distributed in the Afrotropical and Oriental realms, five of which have been recorded from China (WSC 2026) including two from Hunan Province (Yin et al. 2012; Li et al. 2022). Over the past ten years, only a few studies on *Calommata* have been conducted, including one from China (Kim and Lee 2018; Dippenaar-Schoeman et al. 2020; Li et al. 2022; Obae and Kaneda 2025).

During the examination of spider specimens collected from Hunan Province, one new species, *Calommata yueluensis* sp. nov. (♂♀), and the male of *C. hangzhica* Li & Xu, 2022 were identified, which are described here.

## Material and methods

Specimens were stored in 95% ethanol. The left male palps were used for description. The female genitalia were dissected and cleared in lactic acid solution and trypsase. Specimens were measured and photographed using an Olympus BX41 compound microscope. Focus-stack images were generated by Helicon Focus ver. 3.10 and modified by Adobe Photoshop 2021. All measurements are in millimeters. Leg measurements are as follows: total length (femur, patella+tibia, metatarsus, tarsus). The map was created using ArcGIS ver. 10.2 and modified using Adobe Photoshop 2021. All specimens are deposited at the College of Life Sciences, Hunan Normal University (HNU), Changsha, Hunan Province, China. The terminology used in text and figure legends follows Fourie et al. (2011) and Li et al. (2022).

Abbreviations:

**elev.** = elevation **E** = embolus.

**AER** = anterior eye row **MOA** = median ocular area.

**ALE** = anterior lateral eye **PER** = posterior eye row.

**AME** = anterior median eye **PLE** = posterior lateral eye.

**AME–ALE** = distance between **AME and ALEPME** = posterior median eye.

**AME–AME** = distance between **AMEs PME–PLE** = distance between **PME and PLE C** = conductor **PME–PME** = distance between PMEs.

The Universal Genomic DNA Kit was used to extract genomic DNA from muscle tissues of the legs of the male of *Calommata hangzhica* Li & Xu, 2022. Polymerase chain reactions (PCR) were conducted in 25 μl volumes, including 2 μl of genomic DNA, 1 μl of 10 μM forward and reverse primers, 12.5 μl 2×Taq PCR MasterMix (TianGen Biotech, Beijing, China), and 8.5 μl double-distilled H_2_O (Zhou et al. 2025). The PCR primer for a partial fragment of the mitochondrial cytochrome c oxidase subunit I (COI) gene was the universal primer for invertebrate DNA coding LCO1490 and HCO2198 (Folmer et al. 1994). The annealing and extension temperature (Li et al. 2022), as well as all amplifications followed standard protocols (Xu et al. 2015). The PCR products were purified and sequenced in Qingke Biotechnology Company (Changsha, China). The sequencing results were visualized, assembled, edited and aligned in Geneious Prime ver. 2025.0.2. (https://www.geneious.com). All sequences were analyzed using BLAST and deposited in GenBank (PX671534).

Phylogenetic analyses were performed using the COI gene. The ingroup included 14 *Calommata* specimens, one of which represents a novel species. Three *Atypus* specimens and one *Araneus* species were used as the outgroup, with all sequences obtained from GenBank. Phylogenetic analysis was inferred using the maximum likelihood (ML) method based on the Kimura (Kimura 1980) 2-parameter model of nucleotide substitutions. The initial tree for the heuristic search was selected by choosing the tree with the superior log-likelihood between a neighbor-joining (NJ) tree (Saitou and Nei 1987) and a maximum parsimony (MP) tree. Branch lengths were computed using ML (Nei and Kumar 2000) and are presented as the number of substitutions per site. Nodal support was assessed using 1000 bootstrap replicates, and branches with bootstrap support values below 50% were collapsed. The partial deletion option was applied to eliminate all positions with less than 50% site coverage, resulting in a final data set comprising 664 positions. Evolutionary analyses were conducted in MEGA12 (Kumar et al. 2024) utilizing up to eight parallel computing threads. The ML tree was refined using Adobe Photoshop 2021.

## Results

### Taxonomy

**Family: Atypidae Thorell, 1870**

#### Genus Calommata

Taxon classificationAnimaliaAraneaeAtypidae

Lucas, 1837

085052F3-0274-5E26-B62D-EEED8AD3D66B

##### Type species.

*Calommata fulvipes* (Lucas, 1836) from Indonesia.

##### Remarks.

The taxonomic key excludes the female of *Calommata pichoni*, which has been reported from Zhejiang Province (Schenkel 1963). It is documented only in German research, which records morphological analyses, with no images and a detailed description of the epigynal structure. Therefore, it is difficult to construct a reliable taxonomic key based on the existing publication of *C. pichoni*.

### Key to the *Calommata* of China

[males of *C. jinggangica*, *C. pichoni* and *C. yuanjiangica* unknown]

**Table:** 

1	Males	**2**
–	Females	**4**
2	Distal end of embolus overlaps with conductor in ventral view (Fig. 2C, D) (Hunan)	***C. yueluensis* sp. nov**.
–	Between the distal end of embolus and conductor with gap in ventral view	**3**
3	Cheliceral base with a row of small teeth (Fig. 6G; Li et al. 2022, fig. 7d) (Hebei and Hubei)	***C. signata***
–	Cheliceral base with three or four large teeth (Figs 5E, 6F) (Hunan)	***C. hangzhica***
4	Chelicerae with single row of teeth which alternate in size; gap between the middle pair of receptacula relatively wide (Fig. 6C; Li et al. 2022, fig. 7b, c) (Hebei and Hubei)	***C. signata***
–	Chelicerae apical and basal teeth smaller; gap between the middle pair of receptacula relatively narrow	**5**
5	Chelicerae with two small teeth abruptly reduced in size in the midsection of the tooth row (Fig. 6A; Li et al. 2022, fig. 5d, e) (Zhejiang)	***C. hangzhica***
–	Chelicerae with four or five large teeth in the midsection of the tooth row	**6**
6	Receptacula with relatively fewer larger and round granules (Li et al. 2022, fig. 6f) (Hunan and Jiangxi)	***C. jinggangica***
–	Receptacula with relatively more and smaller granules	**7**
7	Thick basal stalks of the receptaculum and the middle pair of receptacula broader and larger (Li et al. 2022, fig. 9f) (Yunnan)	***C. yuanjiangica***
–	Slender basal stalks of the receptaculum and receptacula size show no significant difference in size (Fig. 3B) (Hunan)	***C. yueluensis* sp. nov**.

#### Calommata yueluensis sp. nov.

Taxon classificationAnimaliaAraneaeAtypidae

2E5D6182-E17C-5CB4-9C9E-75812CF25C84

https://zoobank.org/0CDBA161-11B2-47E3-8831-C651C9C2D1CC

Figs 1, 2, 3

##### Type material.

***Holotype*** • male (YLS-17-1005): **China, *Hunan Province*, Changsha City, Yuelu District, Yuelu Mountain**: 28.19013°N, 112.94269°E, elev. 141 m; 13 July 2017; G.C. Zhou leg. ***Paratype*** • 3 females (YLS-16-1212): 28.18129°N, 112.93282°E; elev. 234 m; 08 December 2016; G.C. Zhou leg; • 1 female (YLS-17-0201): 28.18129°N, 112.93282°E; elev. 234 m; 09 February 2017; G.C. Zhou leg; • 1 female (YLS-17-0303): 28.18325°N, 112.93699°E; elev. 163 m; 01 March 2017; G.C. Zhou leg.

**Figure 1.**
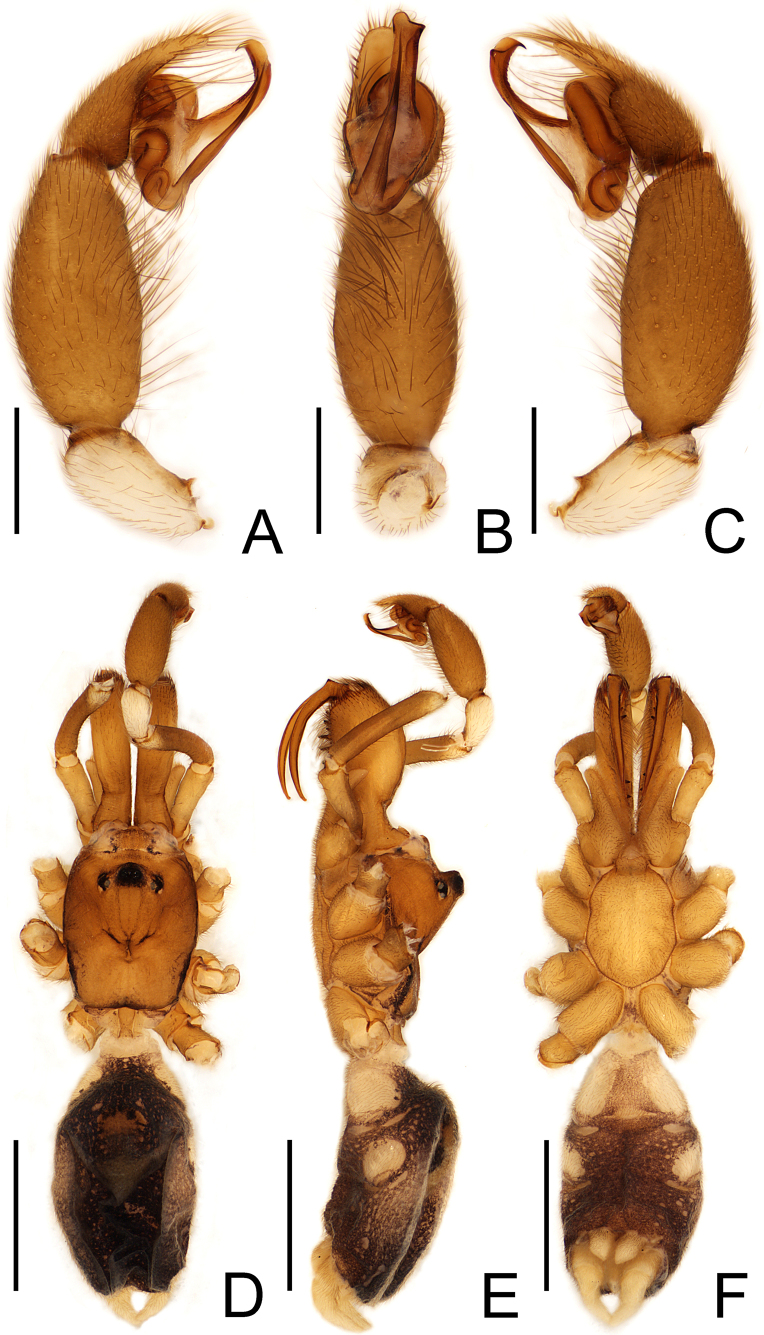
*Calommata yueluensis* sp. nov., left palp of male holotype (**A–C**). **A**. Palp, prolateral view; **B**. Ditto, ventral view; **C**. Ditto, retrolateral view; habitus of male (**D–F**); **D**. Habitus, dorsal view; **E**. Ditto, lateral view; **F**. Ditto, ventral view. Scale bars: 0.5 mm (**A–C**), 2 mm (**D–F**).

**Figure 2.**
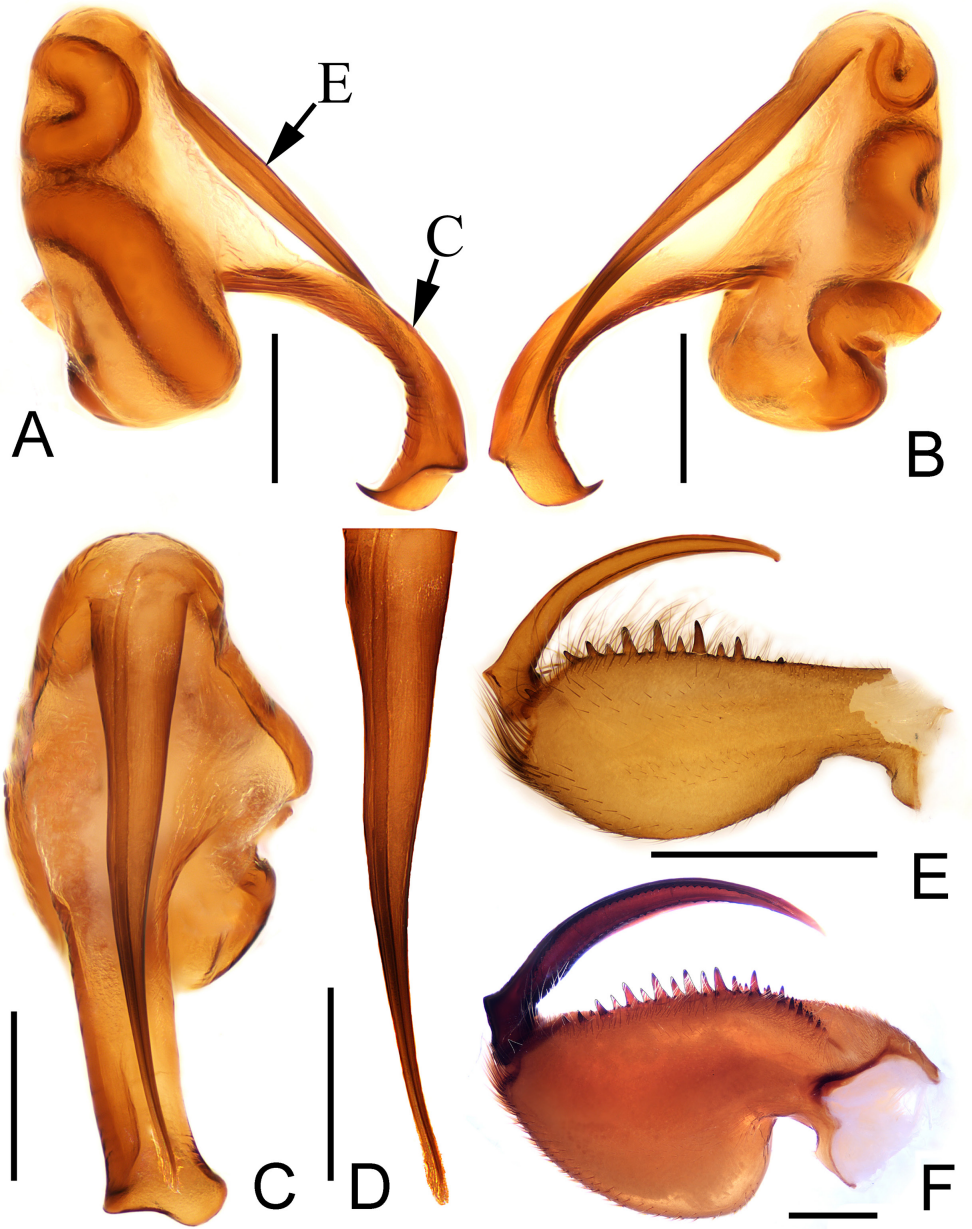
*Calommata yueluensis* sp. nov., genital bulb of left palp (**A–D**). **A**. Genital bulb, prolateral view; **B**. Ditto, retrolateral view; **C**. Ditto, ventral view; **D**. Embolus, ventral view; left chelicerae (**E, F**); **E**. Male chelicerae, inner lateral view; **F**. Female chelicerae, inner lateral view. E = embolus; C = conductor. Scale bars: 0.2 mm (**A–D**), 1 mm (**E–F**).

**Figure 3.**
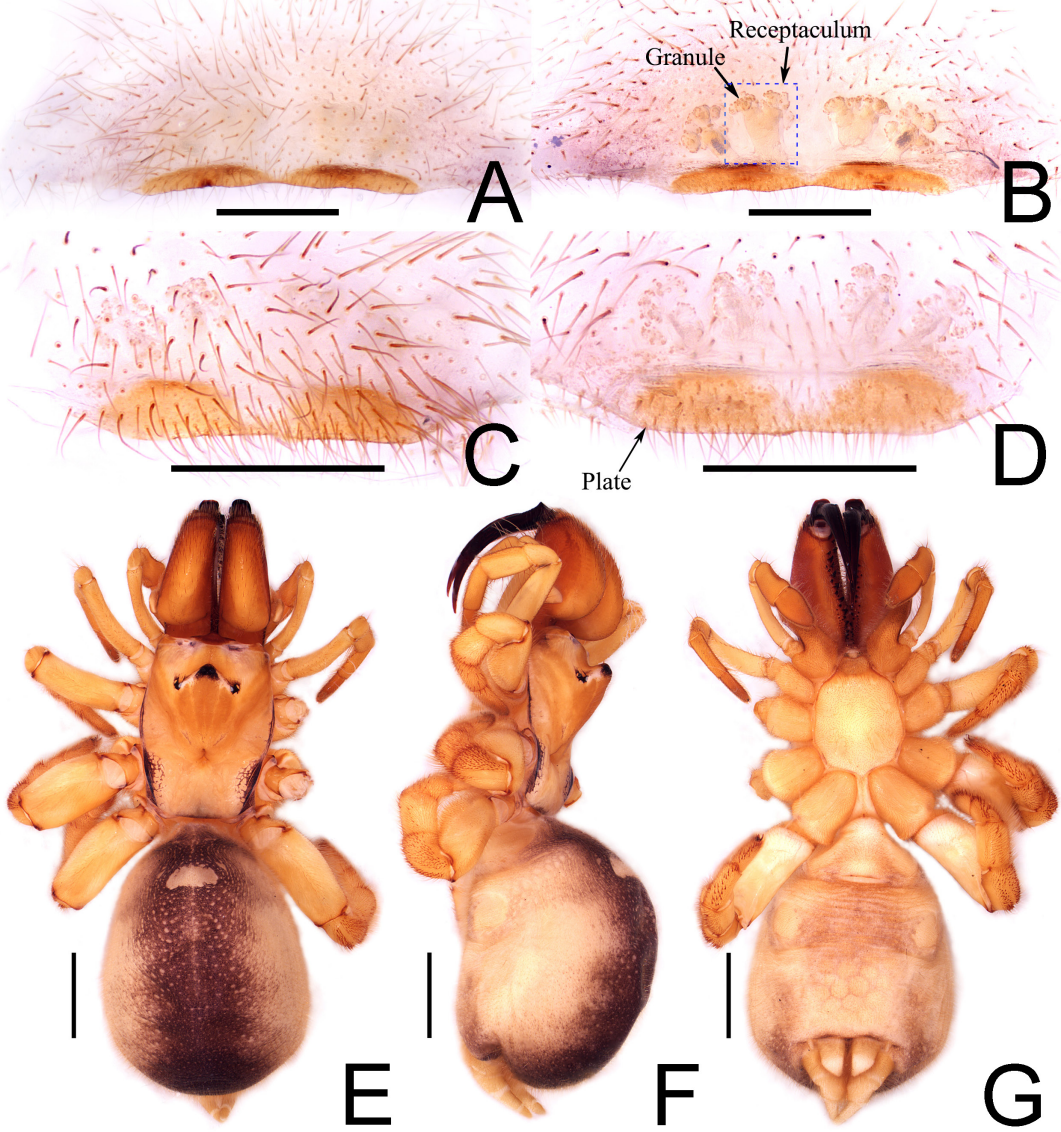
*Calommata yueluensis* sp. nov., genitalia of female paratype (**A, B**). **A**. Epigyne, ventral view; **B**. Vulva, dorsal view; habitus of male (**E–G**); **E**. Habitus, dorsal view; **F**. Ditto, lateral view; **G**. Ditto, ventral view; female (YLS-17-0201) (**C, D**); **C**. Epigyne, ventral view; **D**. Vulva, dorsal view. Scale bars: 0.5 mm (**A–D**), 2 mm (**E–G**).

**Figure 4.**
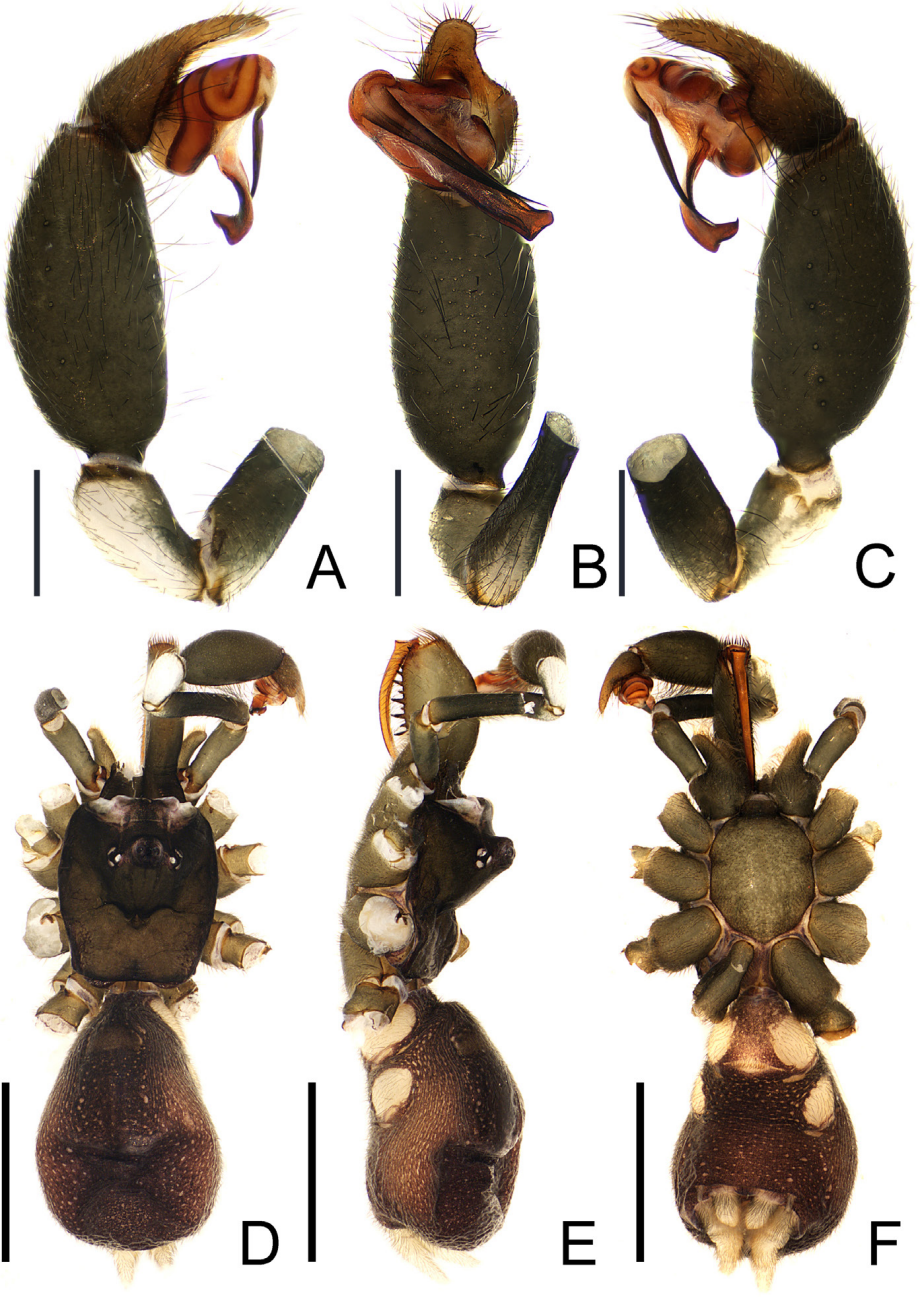
*Calommata hangzhica* Li & Xu, 2022, left palp of male (**A–C**). **A**. Palp, prolateral view; **B**. Ditto, ventral view; **C**. Ditto, retrolateral view; habitus of male (**D–F**); **D**. Habitus, dorsal view; **E**. Ditto, lateral view; **F**. Ditto, ventral view. Scale bars: 0.5 mm (**A–C**), 2 mm (**D–F**).

**Figure 5.**
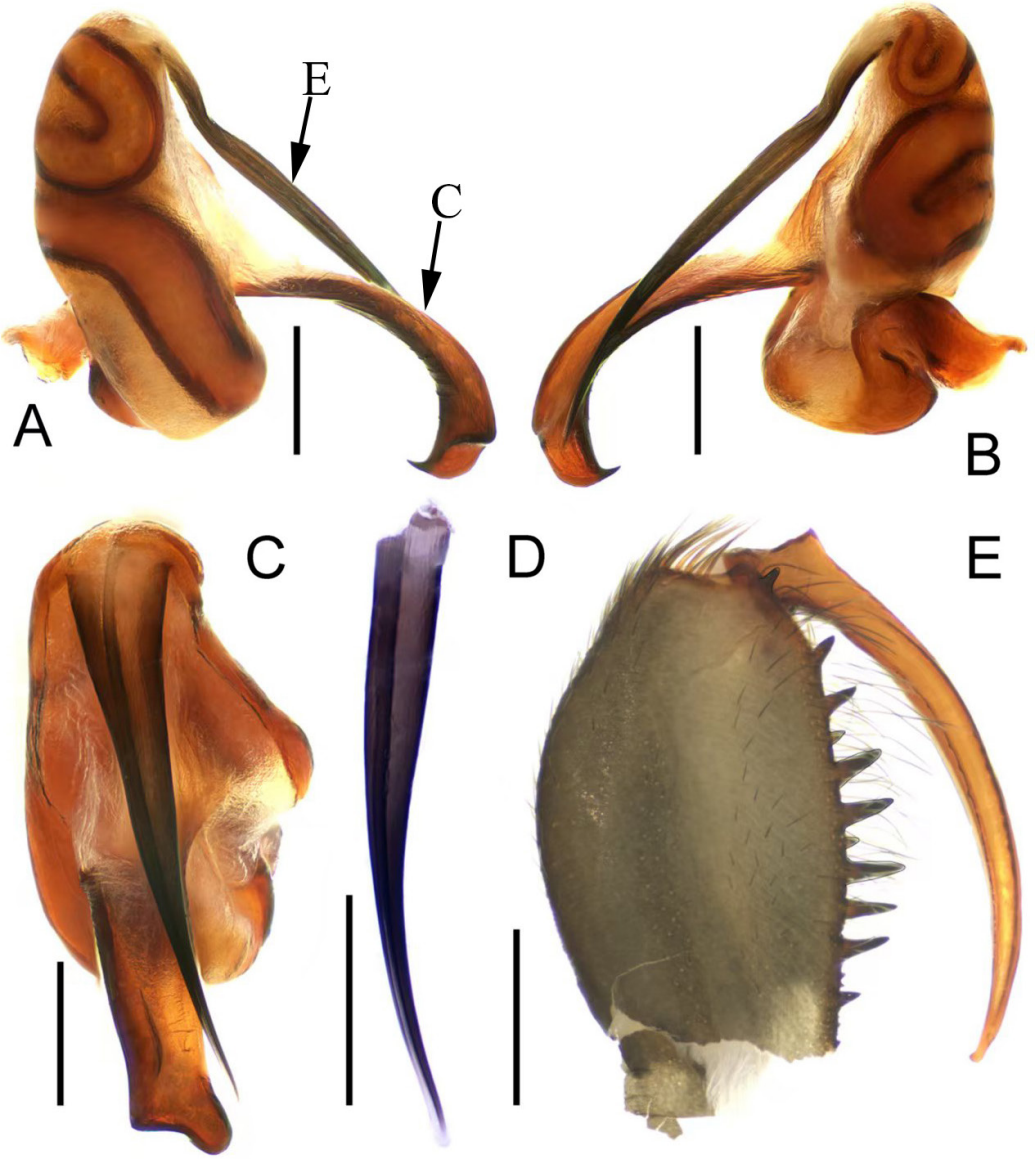
*Calommata hangzhica* Li & Xu, 2022, genital bulb of left pal (**A–D**). **A**. Genital bulb, prolateral view; **B**. Ditto, retrolateral view; **C**. Ditto, ventral view; **D**. Embolus, ventral view; left chelicerae of male; **E**. Chelicerae, inner lateral view. E = embolus; C = conductor. Scale bars: 0.2 mm (**A–D**), 0.5 mm (**E**).

**Figure 6.**
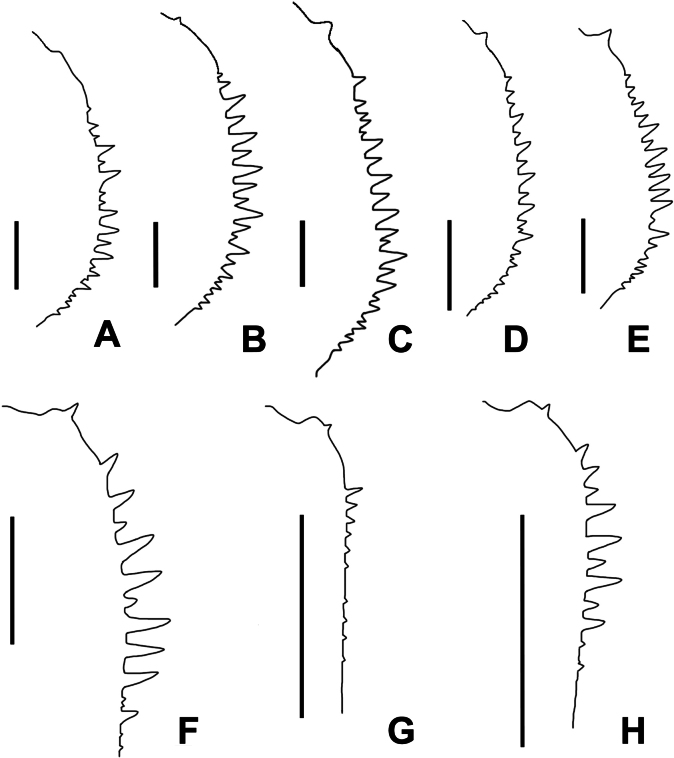
Cheliceral teeth of female (**A–E**), inner lateral view. **A**. *Calommata hangzhica* Li & Xu, 2022; **B**. *C. jinggangica* Li & Xu, 2022; **C**. *C. signata* Karsch, 1879; **D**. *C. yuanjiangica* Li & Xu, 2022; **E**. *C. yueluensis* sp. nov.; cheliceral teeth of male (**F–H**), inner lateral view; **F**. *C. hangzhica*; **G**. *C. signata*; **H**. *C. yueluensis* sp. nov. Scale bars: 0.5 mm (**A, F**), 1 mm (**B–E, G–H**).

##### Etymology.

The specific name refers to the type locality. Adjective.

##### Diagnosis.

The new species resembles *C. signata* Karsch, 1879 in having a similar embolus and female cheliceral teeth (Figs 2, 3A–D, 6G, H; Kim and Lee 2018, fig. 8F–H; Li et al. 2022, fig. 7c, d), but can be differentiated by 1) embolus 2 times longer than conductor in ventral view (Fig. 2C; vs. 3 times longer in *C. signata*); 2) apex of conductor with one prominent tooth on the dorsal surface (Fig. 2A–C; vs. slight tooth in *C. signata*); 3) larger and smaller cheliceral teeth arrange alternately (Figs 2E, 6H; vs. cheliceral teeth decrease gradually from fang base to cheliceral base in *C. signata*); 4) widest gap between the middle pair of receptacula subequal to the height of receptacula (Fig. 3A, B; vs. nearly 1.5 times the height in *C. signata*); 5) slender basal stalks of the receptaculum about 2 times longer than wide (Fig. 3B; vs. squat and about 1.2 times longer than wide in *C. signata*); and 6) receptaculum with more and aggregated granules (Fig. 3B; vs. fewer and scattered in *C. signata*).

##### Description.

**Male** (holotype) (Fig. 1D–F). Total length 6.64 (with chelicerae). Carapace 1.91 long, 1.56 wide; abdomen 2.63 long, 1.68 wide. Carapace square and yellow brown; median ocular region raised and dark; radial groove distinct. Chelicerae with single row of teeth, apical and basal teeth smaller. Sternum, labium and endite yellowish-brown. Eye sizes and interdistances: AME 0.08, ALE 0.11, PME 0.08, PLE 0.09, AME–AME 0.07, AME–ALE 0.18, PME–PME 0.46, PME–PLE 0.03. MOA 0.14 long, anterior width 0.22, posterior width 0.58. Clypeus height 0.61. Leg measurements: leg I 6.79 (1.88, 1.87, 1.57, 1.47), II 6.34 (1.63, 1.57, 1.54, 1.60), III 5.80 (1.44, 1.28, 1.27, 1.81), IV 7.35 (1.51, 1.79, 1.85, 2.20). Leg formula: 4123. Leg light yellowish-brown, weakly covered with bristles; tibiae and metatarsi of legs II–IV covered with spinules. Abdomen dark, with brown scutum anteriorly. Spinnerets light yellow.

Palp (Figs 1A–C, 2A–D). Tibia swollen, about 2 × longer than wide; cymbium yellow and short. Embolus and conductor oriented longitudinally, pointing distally. Conductor with sclerotized margin; apex slightly broadened, with one prominent tooth and sharp edge opposite the tooth. Embolus slender, apex narrower than base with slight bend in ventral view.

**Female** (paratype, Fig. 3E–G). Total length 13.43 (with chelicerae). Carapace 4.08 long, 3.19 wide; abdomen 6.29 long, 4.61 wide. Eye sizes and interdistances: AME 0.11, ALE 0.17, PME 0.18, PLE 0.11, AME–AME 0.10, AME–ALE 0.58, PME–PME 1.07, PME–PLE 0.40. MOA 0.47 long, anterior width 0.30, posterior width 1.23. Clypeus height 0.65. Leg measurements: leg I 6.09 (2.16, 2.10, 1.02, 0.81), II 5.68 (1.83, 1.90, 1.04, 0.91), III 6.47 (2.25, 2.33, 0.96, 0.93), IV 7.23 (2.80, 2.41, 1.13, 0.89). Leg formula: 4312. Legs robust and orange. Carapace orange brown, marginal area with black striations; fovea distinct. Chelicerae with a line of various-sized teeth, cheliceral base with a few rows of tiny denticles. Abdomen with white scutum anteriorly; genital furrow curved. Other characters as in male.

Epigyne (Fig. 3A–D). Female genitalia with two broad and weakly sclerotized basal plates in ventral view. Two cauliflower-like receptacula on each plate in dorsal view; apex with sclerotized particles and basal stalks relatively smooth; middle receptacula larger than the lateral. Female palp short, tibiae and tarsi flattened.

##### Distribution.

Known only from the type locality (Fig. 8).

**Figure 7.**
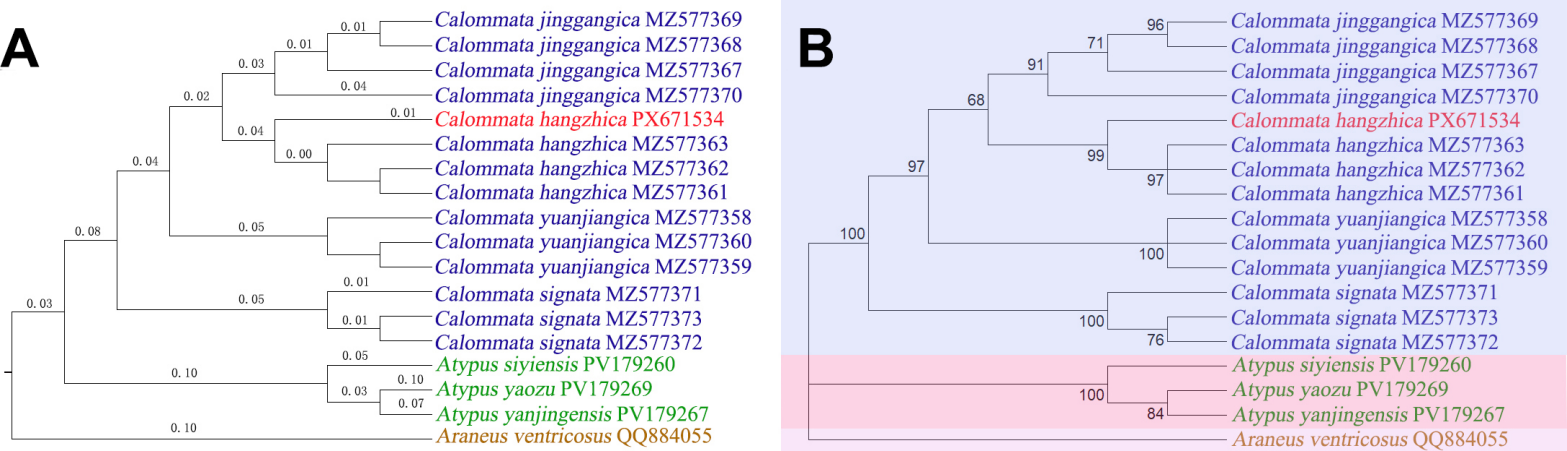
Phylogenetic trees. **A**. Maximum likelihood tree (K2P model), the number of substitutions per site is shown above the branches; **B**. Bootstrap consensus tree, bootstrap support values (ML) are shown next to the branches.

**Figure 8.**
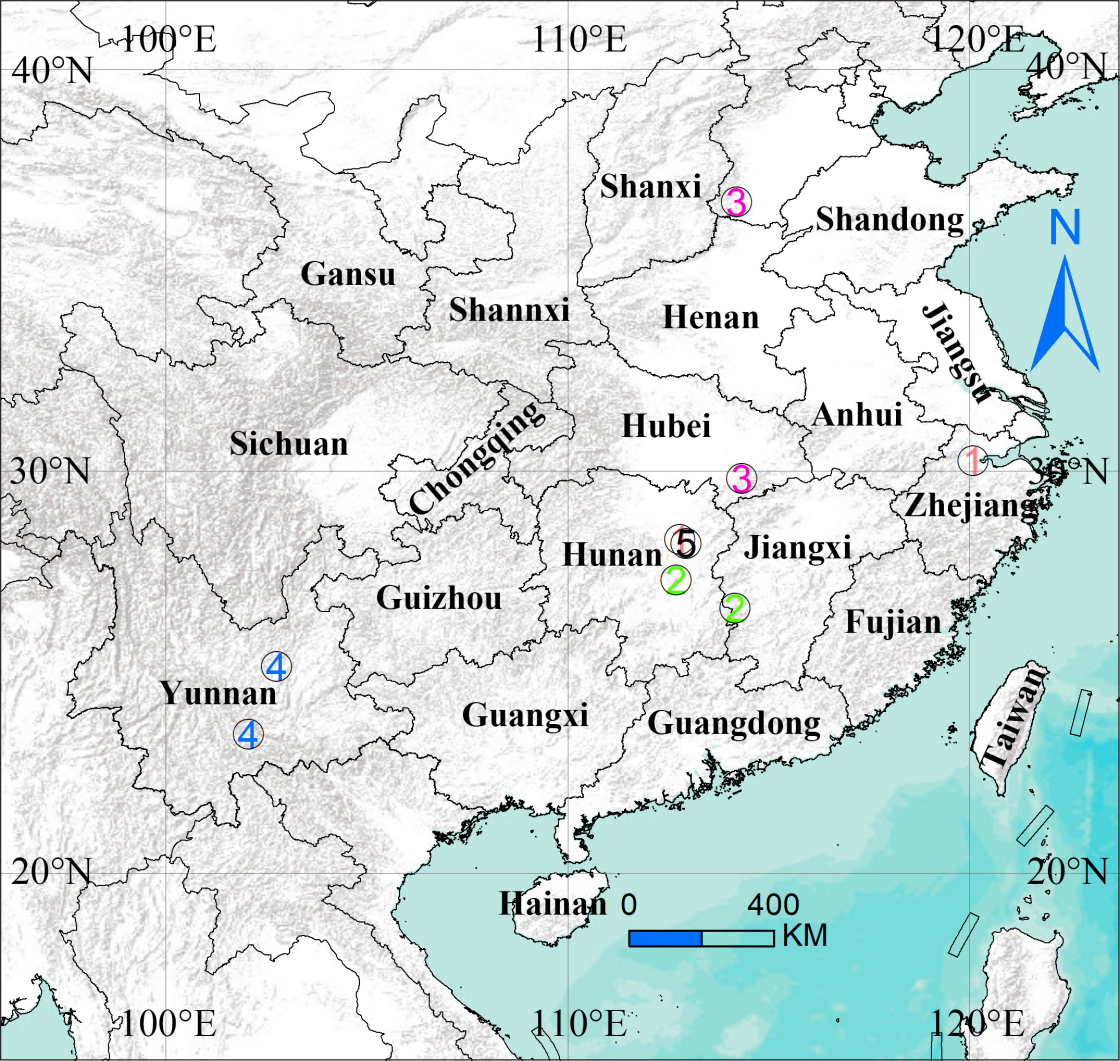
Distribution of *Calommata* of China: 1 *C. hangzhica* Li & Xu, 2022; 2 *C. jinggangica* Li & Xu, 2022; 3 *C. signata* Karsch, 1879; 4 *C. yuanjiangica* Li & Xu, 2022; 5 *C. yueluensis* sp. nov.

##### Variation in female genitalia.

The size of the receptaculum and plate varies in the examined samples (Fig. 3A–D), probably due to differences in female age. The receptaculum seems to be well developed in specimen YLS-16-1212 (Fig. 3A, B) and less developed in specimen YLS-17-0201 (Fig. 3C, D).

##### Remarks.

We tried to extract DNA from *Calommata yueluensis* sp. nov. (♂♀). Unfortunately, the quality of the DNA extracted from these specimens was not good enough for PCR, possibly because they had been deposited for nearly 10 years and their DNA had not been adequately preserved. However, through thorough morphological analyses and comparisons, it is confirmed that the specimen is distinct from all known *Calommata* species to date, and is hereby recognized as a new species.

#### Calommata hangzhica

Taxon classificationAnimaliaAraneaeAtypidae

Li & Xu, 2022

95AB86E3-E80C-52BD-83A5-A44D05327C07

Calommata hangzhica Li & Xu, 2022: appendix S1, pl. 1, fig. 5c–f (♀).

##### Material examined.

• 1 male (HNU-WS-250828): China, Hunan Province, Changsha City, Wangcheng District, Wushan Forest Park; 28.29321°N, 112.79764°E; elev. 86 m; 28 August 2025; X.J. Peng, S.L. Li, W.L. Yan, C.P. Zou and F.Q. Wu leg.

##### Diagnosis.

The male of *Calommata hangzhica* Li & Xu, 2022 resembles that of *C. yueluensis* sp. nov. in having a similar embolus (cf. Figs 2, 5), but can be distinguished by 1) embolus and conductor directed obliquely and retrolaterally towards endites in ventral view (Figs 1B, 4B; vs. directed vertically and retrolaterally towards apex of chelicerae); 2) apex of embolus separates with conductor by a gap in ventral view (Fig. 5C; vs. overlaps with conductor in *C. yueluensis*); and 3) cheliceral base with three or four large teeth (Figs 5E, 6F; vs. with several small teeth in *C. yueluensis*).

##### Description.

**Male**. (HNU-WS-250828) (Fig. 4D–F). Total length 6.65 (with chelicerae). Carapace 2.09 long, 1.64 wide; abdomen 2.90 long, 2.12 wide. Carapace square and brown in colour; median ocular region strongly raised; fovea and radial groove distinct. Chelicerae dark brown with 13 teeth arranged in a single row. Sternum and labium brown; endite greatly enlarged. Eye sizes and interdistances: AME 0.08, ALE 0.09, PME 0.07, PLE 0.06; AME–AME 0.06, AME–ALE 0.25, PME–PME 0.52, PME–PLE 0.03. MOA 0.22 long, anterior width 0.25, posterior width 0.65. Clypeus height 0.48. Leg measurements: leg I 7.85 (2.29, 2.05, 1.81, 1.70), II 7.19 (1.79, 1.82, 1.84, 1.74), III 7.08 (1.71, 1.51, 1.76, 2.10), IV 8.40 (2.33, 1.83, 1.94, 2.30). Leg formula: 4123. Leg yellow brown, weakly covered with bristles; tibiae and apex of metatarsi covered with spurs. Abdomen dark brown, with brown scutum anteriorly. Spinnerets white.

Palp (Figs 4A–C, 5A–D). Tibia swollen, about 2 × longer than wide; cymbium short and light brown. Embolus and conductor oriented obliquely, pointing retrolaterally. Conductor with sclerotized margin, gradually twisted and broadened apex with one prominent small tooth and obtuse edge opposite the tooth. Embolus straight, base slightly curved in lateral view, with wide gap between conductor and embolus.

##### Distribution.

Hunan (Changsha) and Zhejiang (Hangzhou) Provinces, China (Fig. 8).

##### Remarks.

The female of *C. hangzhica* has been reported from Zhejiang Province (Hangzhou) (Li et al. 2022). The specimen collected from Hunan is assigned to *C. hangzhica* based on the following reasons. First, this species is distinct from other described species, based on morphological analyses and comparisons. More importantly, we tried to extract DNA from the male specimen in the present study, and it was found that the smallest pairwise genetic distances between the female *C. hangzhica* specimen and the male specimen were only 0.90% (K2P) and 0.89% (p-distance), which are much smaller than the interspecific distances. Phylogenetic analyses (Fig. 7) further support the identification of the male specimen in the present study as *C. hangzhica*.

##### GenBank accession code.

COI for examined male specimen (HNU-WS-250828): PX671534.

## Supplementary Material

XML Treatment for Calommata

XML Treatment for Calommata yueluensis

XML Treatment for Calommata hangzhica

## Additional information

### Artificial Intelligence (AI) use

The authors accept full responsibility for the content of the manuscript, including the disclosure of any use of AI.

No AI tools were used in the preparation of this manuscript.

### Author ORCIDs

F. Wu https://orcid.org/0009-0000-1749-0606

W. Yan https://orcid.org/0009-0001-9663-9436

G. Zhou https://orcid.org/0000-0002-5757-0317

X. Peng https://orcid.org/0000-0002-2614-3910

### Data availability

All of the data that support the findings of this study are available in the main text.
